# Structure-Activity Relationships of *closo*- and *nido*-Carborane Erlotinib Analogs: Lipophilicity as a Key Modulator of Anti-Glioma Activity

**DOI:** 10.3390/ph18111753

**Published:** 2025-11-18

**Authors:** Belén Dávila, Pablo Vignolo, Martina Silvarrey, Andrés Benítez, Juliana González Schmidt, Carmela de Arteaga Guidotti, María Fernanda García, Hugo Cerecetto, Marcos Couto

**Affiliations:** 1 Grupo de Química Orgánica Medicinal, Instituto de Química Biológica, Facultad de Ciencias, Universidad de la República, Montevideo 11400, Uruguay; bdavila@fcien.edu.uy (B.D.); pvignolo@fcien.edu.uy (P.V.); martinasilvarrey@fcien.edu.uy (M.S.); abenitez02@fcien.edu.uy (A.B.); juliana.gonzalez@fcien.edu.uy (J.G.S.); cdearteaga@fcien.edu.uy (C.d.A.G.); hcerecetto@cin.edu.uy (H.C.); 2 Graduate Program in Chemistry, Facultad de Química, Universidad de la República, Montevideo 11400, Uruguay; 3 Área de Radiofarmacia, Centro de Investigaciones Nucleares, Facultad de Ciencias, Universidad de la República, Montevideo 11400, Uruguay; mfgarcia@fcien.edu.uy; 4 Instituto de Investigación Una Salud, Universidad de la República, Montevideo 11600, Uruguay

**Keywords:** carborane, R_M_^0^, fragmentary log*P*, activity relationship

## Abstract

**Background/Objectives**: To enhance the anti-glioma activity of erlotinib, we previously developed a series of carborane-based analogs exploiting the concept of three-dimensional bioisosterism. These carboranes generally exhibited improved cytotoxicity against glioma cell lines compared with the parent compound erlotinib and additionally showed varying degrees of EGFR inhibition. Given the well-described influence of lipophilicity on pharmacological properties, we aimed to determine this parameter for the new analogs and explore its correlations with biological behaviors. **Methods**: Lipophilicity was assessed experimentally, through chromatographic procedure, in terms of R_M_^0^ and theoretically via fragment-based log*P* calculations (flog*P*) using Hansch–Fujita hydrophobic parameters π of some substituents and the experimentally determined log*D_n_*_-octanol/buffer(7.4)_ of 4-chloro-6,7-bis(2-methoxyethoxy)quinazoline. Additionally, the electronic properties of the carborane clusters were considered using the NMR chemical shifts of cluster carbon-bound protons. **Results**: For the series of carboranes, the R_M_^0^ discretely correlated to the flog*P*. Neither R_M_^0^ nor flog*P* correlated with the electronic characteristics of the carboranes. From the correlation between R_M_^0^ and flog*P*, it was possible to estimate the π value for a *nido*-carboranyl substituent. Cytotoxicities, against glioma cells, exhibited a parabolic dependence on lipophilicity, finding optimal flog*P* for each cellular system. Some tendencies were observed between EGFR inhibition and flog*P*, requiring more hydrophilic compounds for optimal wild-type EGFR inhibition or a specific flog*P* for mutant EGFR inhibition. It was observed that the electronic features of the boron cluster also influenced both biological activities studied. **Conclusions**: Unlike our previous reports, which focused on the synthesis and biological evaluation of carborane-erlotinib analogs, this study establishes for the first time the correlation of lipophilicity and electronic features with cytotoxic and EGFR-inhibitory activities, providing new insights into their structure–activity relationships.

## 1. Introduction

Glioblastoma is a primary malignant brain tumor characterized by a high degree of malignancy and an extremely poor prognosis. The limited efficacy of current treatments underscores the urgent need for novel therapeutic strategies [1,2,3]. Receptors with tyrosine kinase activity (TKRs), such as epidermal growth factor receptor (EGFR), platelet-derived growth factor receptor (PDGFR), or human epidermal growth factor 2 receptor (HER2), play a crucial role in cell growth and differentiation, and their overexpression or mutation is implicated in the development of several cancers [4]. Tyrosine kinase inhibitors, including erlotinib (**Erl**), sunitinib (**Sun**), and lapatinib (**Lap**), have been extensively studied for the treatment of glioblastoma, one of the most aggressive primary brain tumors [5,6,7].

Carborane and other boron-containing clusters have recently gained increasing attention in medicinal chemistry as versatile pharmacophoric motifs. Their well-defined three-dimensional architecture, exceptional stability, and capacity to modulate lipophilicity and electronic distribution make them valuable tools for tuning biological interactions. Several recent reviews have highlighted the expanding role of carborane and boron-based compounds in drug discovery and pharmacological research [8,9,10,11,12].

In previous work, our group developed hybrid compounds by integrating TKR pharmacophores with boron-rich scaffolds, such as *closo*-carboranes, aiming to enhance anticancer activity and explore potential applications in boron neutron capture therapy (BNCT) [13,14,15,16,17]. Among these, Erl-based hybrids demonstrated promising cytotoxicity against high-grade glioma in vivo [18]. For this reason, we recently described a new approach using **Erl** as a structural template [19], in which we explored non-classical bioisosterism by replacing the planar 2D *meta*-ethynylphenylamino group of **Erl** with various carboranyl moieties, serving as three-dimensional (3D) bioisosters. In general, the new **Erl** analogs, **1**–**7**, displayed improved cytotoxic activities compared with the parent compound against several glioma cell lines, with favorable selectivity indexes (Table 1).

In the present study, we focus on assessing lipophilicity and electronic features and correlating these parameters with cytotoxicity and EGFR-inhibitory activity, providing new insights into structure–activity relationships.

From the extrathermodynamic approach developed by Corwin Hansch [20,21], it has been assumed that the physicochemical properties of a molecule correlate with its biological activity, with lipophilicity being one of these properties. This descriptor is essential for a molecule to cross cell membranes, interact with its molecular target, distribute within the body, undergo metabolic transformation, and be excreted, thereby directly affecting its overall biological response.

Herein, we describe the study of the lipophilic properties of carborane-containing **Erl** analogs **1**–**7** and examine their correlation with cytotoxicity against glioma cells and EGFR-inhibitory activity. Our findings reveal that this molecular property plays a significant role in modulating the biological activity of these analogs.

## 2. Results and Discussion

### 2.1. Lipophilicity Descriptors

#### 2.1.1. Determination of R_M_^0^ and flog*P*

Two different approaches were assayed to determine the lipophilicity descriptor of each molecule. On the one hand, we used reverse phase thin-layer chromatography (RPTLC) to determine the descriptor R_M_^0^ [22,23,24], and on the other hand, we calculated the fragmentary lipophilicity, flog*P*, employing an experimental log*P* determined by the shake-flask method. Regarding R_M_^0^ (Table 2), the values were clearly correlated to the structure of compounds. For instance, analog **4**, which bears an ionic decapped-carboranyl (*nido*-carboranyl) substituent, was the most hydrophilic analog (R_M_^0^ = 1.46), while analog **5**, containing two quinazolinyl moieties, exhibited the highest lipophilicity (R_M_^0^ = 3.94). To calculate flog*P* (Table 2), we employed as starting data the log*P* determined experimentally by partition between *n*-octanol and water and as log*D*_7.4_ of 4-chloro-6,7-bis(2-methoxyethoxy)quinazoline (log*D*_7.4_ = 1.023) and the corresponding Hansch-Fujita hydrophobic parameters π [25,26,27]. Except for the decapped-carboranyl moiety, the remaining π parameters for carboranyl groups have been previously described [26,27]. Similarly to R_M_^0^, flog*P* values correlated well with analog structures. For example, among the *meta*-carborane derivatives, analog **6**, bearing a polar NH connector between the quinazolinyl and carboranyl fragments, is more hydrophilic (flog*P* = 2.64) than analog **2** (flog*P* = 4.92).

Importantly, the differences in flog*P* among the *ortho*-, *meta*-, and *para*-carborane derivatives can be directly rationalized by the dipole moment of the carborane cage [28]. As the dipole moment decreases, the overall hydrophobicity of the compound increases. For example, the *para*-carborane (1,12-isomer) **3** exhibits the lowest dipole moment and correspondingly the highest lipophilicity (flog*P* = 5.10), whereas *ortho*- and *meta*-isomers, **1** and **2**, respectively, display higher dipole moments and reduced lipophilicity. This trend is observed consistently across both series with and without polar NH linkers, highlighting the dominant role of the cage dipole in modulating lipophilicity.

#### 2.1.2. Correlation Between R_M_^0^ and flog*P*

**Figure 1:**
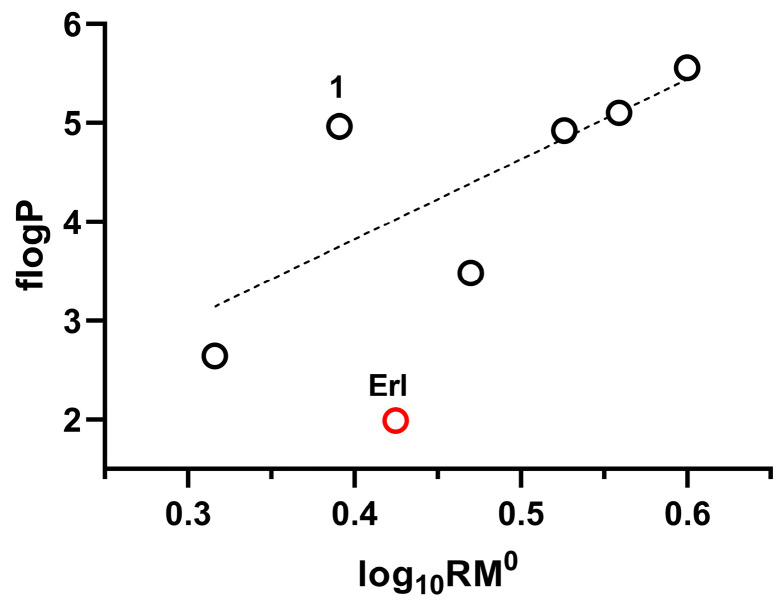
Correlation between the log_10_R_M_^0^ and flog*P*. The red data point corresponds to erlotinib (**Erl**), which was not included in the regression analysis. The black circles correspond to the individual carborane-based analogs, plotted according to the experimental values reported in Table 1 and Table 2.

The R_M_^0^ values determined in this work, expressed as log_10_R_M_^0^, were subjected to linear regression against flog*P* values for the full set of compounds (**1**–**3**, **5**–**7**, and **Erl**). A moderate correlation was obtained (*r* = 0.6955, *r*_adj_ = 0.6168, *RSS* = 5.7616). When **Erl** was excluded, restricting the analysis to the carborane-based analogs (**1**–**3** and **5**–**7**), the linear regression was improved (*r* = 0.7718, *r*_adj_ = 0.7032, *RSS* = 2.5625), although compound **1** emerged as an outlier (Figure 1). Considering that compound **1** has the most acidic proton, on the carbon at the vertices of the boron clusters (see values of δ_H_ as an indicator of the acidity, Table 2) [29] and that this phenomenon could affect the lack of a good relationship between log_10_R_M_^0^ and flog*P*, it was removed from the analysis, achieving an improvement in the correlation (*r* = 0.9599, *r*_adj_ = 0.9462, *RSS* = 0.4724). This greater acidity of the C-H could lead to an overestimation of the hydrophilicity of analog **1** determined herein by the RPTLC method (R_M_^0^), where the mobile phases are mixtures of water and methanol. This overestimation could be true since this type of hydrogen, mainly in the *ortho*-carboranyl system, has been shown to interact by hydrogen bonding with oxygen-bearing acceptors [30,31], as is the case with our mobile phases. In contrast, the fragmentary values π used to calculate flog*P*, which were also determined by chromatographic methods (RPHPLC), employed an acidic mobile phase (methanol–aqueous 0.1 M phosphoric acid). In this mobile phase, the impact of the C-H acidity, and therefore the hydrophilicity overestimation, may not be as evident.

#### 2.1.3. Approaching the Hansch–Fujita Hydrophobic Parameter π for the *nido*-Carboranyl Substituent

From the correlation between log_10_R_M_^0^ and flog*P* (Section 2.1.2, using analogs **1**–**3** and **5**–**7**) and the log_10_R_M_^0^ of analog **4**, which ports the *nido*-carboranyl substituent, it was possible to estimate the Hansch–Fujita π parameter for this moiety. The resulting value (π = 1.3) reflects the markedly increased hydrophilicity of the decapped-carboranyl moiety compared with its *o*-carboranyl counterpart (non-deboronated analog system) (π*_o_*_-carboranyl_ = 4.33 [27]). This difference reflects the ionic character of the *nido*-carboranyl substituent, which significantly enhances its affinity for aqueous environments.

### 2.2. Structure–Activity Relationships

#### 2.2.1. Correlations Between Lipophilicity and Cellular Cytotoxicity

Observing the lipophilicities and the IC_50_ against the different cells (Table 1), we anticipated that these parameters could be quadratically correlated. For example, against U87 MG glioma cells, the least active compounds, analogs **4** and **5**, possess flog*P* of 1.91 (calculated from the correlation between R_M_^0^ and flog*P*, Section 2.1.2) and 5.56 (Table 2), respectively, while one of the most active, analog **7**, possesses a flog*P* of 3.48 (Table 2). Therefore, we analyzed, for the complete population of compounds (**1**–**7** and **Erl**), data fits to parabolic equations by regression analysis. Such parabolic rather than linear relationships are commonly observed because biological activity depends on a balance between aqueous solubility and membrane permeability. Compounds with low lipophilicity are generally more soluble but less permeable, while highly lipophilic analogs tend to cross membranes more efficiently but suffer from reduced solubility or non-specific binding. Consequently, an intermediate log*P* value typically results in optimal cytotoxicity.

**Figure 2:**
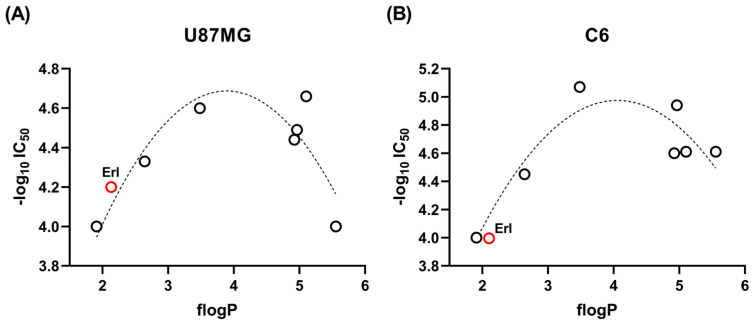
Correlation between the flog*P* and cellular cytotoxicity. (**A**) Against human U87 MG glioma cells. (**B**) Against murine C6 glioma cells. The red data point corresponds to erlotinib (**Erl**), which was not included in the regression analysis. The black circles correspond to the individual carborane-based analogs, plotted according to the experimental values reported in Table 1 and Table 2.

In the case of U87 MG cells, a clear quadratic distribution was observed between the cytotoxic effects and the molecular lipophilicity, expressed as flog*P* (*r* = 0.8656, *r*_adj_ = 0.8055, *RSS* = 0.1137) (Figure 2A). In the case of carborane population, it resulted in improvement in the *r* and *RSS* values (*r* = 0.8740, *RSS* = 0.1017), although *r*_adj_ decreased slightly (*r*_adj_ = 0.8037). For these cells, and with the study population, the lipophilicity, expressed as flog*P*, for optimal activity is 3.9.

For F98 cells, a quadratic distribution was obtained between the cytotoxic effects and the molecular lipophilicity but was statistically weaker than in the case of U87 MG (*r* = 0.6938, *r*_adj_ = 0.5234, *RSS* = 0.4687). However, when analog **4**, whose flog*P* was estimated (from the correlation between R_M_^0^ and flog*P*, as described in Section 2.1.2), was removed from the analysis, the statistics improved (*r* = 0.8561, *r*_adj_ = 0.7742, *RSS* = 0.2394). For these cells, and with the complete study population, the lipophilicity, expressed as flog*P*, for optimal activity is 4.5.

For the other murine glioma cell line, C6, the best results were obtained in statistical terms, observing a robust quadratic correlation between the cytotoxic effects and the molecular lipophilicity with the entire population of compounds under study (*r* = 0.9287, *r*_adj_ = 0.8985, *RSS* = 0.1438) (Figure 2B). For these cells, and with the study population, the lipophilicity, expressed as flog*P*, for optimal activity is 4.1.

Finally, for the healthy glial cells, a mix of primary glial cells (Table 1), it was also observed that the cytotoxic effects are quadratically correlated with the molecular lipophilicities of the studied compounds (*r* = 0.8749, *r*_adj_ = 0.8196, *RSS* = 0.0283). For these cells, and with the study population, the lipophilicity, expressed as flog*P*, for maximum toxicity is 4.0.

The regression analyses consistently indicated that the most favorable cytotoxic activities were associated with intermediate lipophilicity values, with optimal flog*P* ranging from 3.9 (U87 MG) to 4.5 (F98) depending on the tumor cell type. Notably, a comparable optimum flog*P* (≈4.0) was also observed for non-tumor glial cells, suggesting that the relationship between lipophilicity and cytotoxicity is not tumor-specific. These results imply that future analog design should carefully modulate lipophilicity within this relatively narrow window (≈3.9–4.5) to enhance the likelihood of achieving activity against glioma cells, while additional structural features will be required to improve tumor selectivity and reduce cytotoxicity toward healthy glia.

#### 2.2.2. Correlations Between Lipophilicity and EGFR Inhibitions

**Figure 3:**
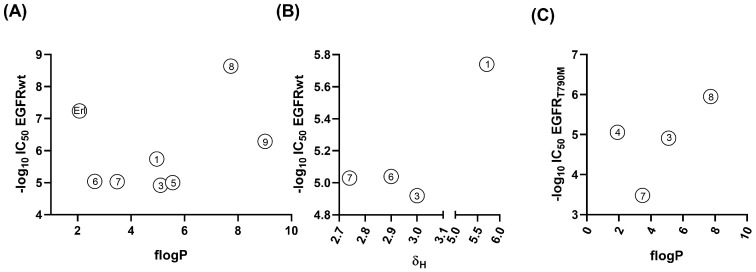
(**A**) Plot of fLog*P* and wild-type EGFR inhibition for compounds **1**, **3**, **5**–**9**, and **Erl**. (**B**) Plot of δ_H_ and wild-type EGFR inhibition for carborane-containing **Erl** analogs **1**, **3**, **6** and **7**. (**C**) Plot of fLog*P* and EGFR_T790M_ inhibition for analogs **3**, **4**, **7**, and **8**.

Wild-type EGFR inhibition (Table 1) showed a quadratic relationship with lipophilicity (*r* = 0.7845, *r*_adj_ = 0.5992, *RSS* = 1.4929), although this trend appeared to be driven primarily by the high inhibitory activity of analog **1** (Figure 3A). The artifact that this analog promotes could be due to its capability, as mentioned above, to establish other types of interactions (via the acidic hydrogen bound to the carbon atom located at the vertex of the boron cluster) with EGFR, a phenomenon not necessarily associated with lipophilicity. From the data plotted in Figure 3A, it could be observed that there is a tendency where more hydrophilic compounds, like Erl, are better EGFR inhibitors, indicating that these compounds would be located at the site where the polar ATP is anchored. In this context, analog **1**, the most potent wild-type EGFR inhibitor among the boron-cluster derivatives, is likely to engage in some form of polar interaction. This behavior could be rationalized by the higher acidity of the cluster-bound hydrogen (as observed when plotting δ_H_ versus EGFR inhibition, Figure 3B), which allows analog 1 to establish some type of ionic interaction with the biomolecule. To slightly expand the dataset, two previously reported hybrid derivatives (compounds **8** and **9**) [13] were included in the analysis. Their fragmental lipophilicity values (fLog*P* = 7.73 and 9.01, respectively) were recalculated using the same approach applied to the current series. Interestingly, these compounds also followed the overall tendency, and the highly lipophilic compound **8** exhibited a remarkable potency, being approximately ten times more active than erlotinib. Nevertheless, the comparison between these hybrids and the current series (**1**–**7**) should be made with caution, since the former possess different linker polarity and conformational flexibility, as they were originally designed as hybrids rather than bioisosteric analogs of erlotinib.

For mutant EGFR inhibition data (Table 1), the dataset was too limited to establish a statistically validated correlation. However, a parabolic tendency could be observed (Figure 3C), where analog **7**, with a fLog*P* of 3.48, displayed the best inhibition ability among the current series of compounds. In this case, compound **8** was also incorporated into the analysis, showing activity in line with its high lipophilicity, but the inclusion did not significantly alter the overall trend. These results should therefore be considered preliminary, and additional studies with a larger and more homogeneous series of analogs will be required to draw robust conclusions.

#### 2.2.3. Towards the Inclusion of the Electronic Effects of Boron Clusters in the Correlations

Although the number of carborane-Erl analogs studied was insufficient to investigate the existence of multivariate correlations, we decided to include an electronic parameter of the boron clusters into the analysis to assess potential relationships involving more than one variable. Specifically, we considered the NMR chemical shift of the protons located on the carbons at the vertices of the boron clusters (δ_H_, Table 2) (Section 2.1.1). These chemical shifts, as we saw previously for other systems [32,33,34,35], could be a good descriptor of the electronic density of the different clusters. For the total population of analogs studied, these independent variables, flog*P* and δ_H_, are orthogonal (*r* = 0.476).

The following equations were obtained by the addition of the electronic descriptor, δ_H_ (Table 2):

−log_10_IC_50,U87 MG_ = 2.62 (±0.65) − 0.04 (±0.06) δ_H_ + 1.0 (±0.4) flog*P* − 0.11 (±0.05) flog*P*^2^ *r* = 0.9453, *r*_adj_ = 0.8567, *RSS* = 0.0298, *F* = 5.60 (1)

−log_10_IC_50,F98_ = 3.46 (±1.31) − 0.17 (±0.11) δ_H_ + 0.75 (±0.79) flog*P* − 0.08 (±0.11) flog*P*^2^ *r* = 0.8101, *r*_adj_ = 0.3751, *RSS* = 0.1208, *F* = 1.27 (2)

−log_10_IC_50,C6_ = 0.73 (±0.92) + 0.09 (±0.08) δ_H_ + 2.1 (±0.5) flog*P* − 0.27 (±0.08) flog*P*^2^ *r* = 0.9580, *r*_adj_ = 0.8913, *RSS* = 0.0591, *F* = 7.44 (3)

−log_10_IC_50,mix glial_ = 3.53(±0.09) − 0.028(±0.008)δ_H_ + 0.35(±0.06)flog*P* − 0.037(±0.008)flog*P*^2^ *r* = 0.9949, *r*_adj_ = 0.9872, *RSS* = 0.0006, *F* = 64.97 (4)

Although the standard errors of the electronic descriptor, δ_H_, in Equations (1)–(3) were not good enough, some general conclusions can be mentioned. In the case of the glioblastoma cells U87 MG and F98, the electronic effect negatively impacts the analog cytotoxicity. For example, analog **1** with the most acidic C_cluster_-H (highest δ_H_, Table 2) displayed lower cytotoxicities against these glioblastoma cells (Table 1) than analog **7**, one of the most active compounds (Table 1) with one of the least acidic C_cluster_-H (lowest δ_H_, Table 2). While for the glioblastoma cell C6, the electronic effect has a positive effect on the analog-cytotoxicity (compare activities of analogs **1** and **4**, Table 1).

For the mix of primary glial cells, the tendency was consistent with that described in Equations (1) and (2), with Equation (4) showing greater statistical robustness. However, the interpretation in this case is different. For an anti-tumor agent, higher selectivity implies lower toxicity toward the primary glial cell mixture, and Equation (4) can be used to guide this aspect. In Equation (4), the electronic effect showed a negative contribution; however, to achieve selectivity, its influence should be opposite to that described in the previous paragraph for U87 MG and F98 cells, which is consistent with the experimental biological data (Table 1). For example, when comparing analogs **1** and **3**, the former, less toxic to the primary glial cell mixture, has the highest *δ*_H_ value of the pair (Table 2). Similarly, between analogs **6** and **7**, the less toxic compound (analog **6**) also displays the highest *δ*_H_ value (Table 2).

## 3. Materials and Methods

### 3.1. Chemical Compounds and Cell Lines

**Erlotinib** was purchased from Hong Kong Guokang Bio Technology Co. (Baoji, China). Compounds **1**–**7** were prepared according to reference [13], and compounds **8**–**9** were obtained as described in reference [7]. The human malignant glioblastoma cell line U-87 MG (HTB-14™, ATCC^®^) and the rat astrocytoma-derived cell line C6 (ATCC^®^ CCL-107™) were obtained from the American Type Culture Collection (ATCC, Virginia City, NV, USA). F98 rat glioma cells, histologically characterized as an anaplastic astrocytoma, were a kind gift from María Alejandra Dagrosa, researcher at the Comisión Nacional de Energía Atómica, Argentina.

### 3.2. R_M_^0^ Determination [22]

Reverse-phase TLC experiments were performed on precoated TLC plates SIL RP-18 W/UV254 (Macherey-Nagel GmbH, Düren, NRW, Germany) and eluted with six different concentrations of MeOH (Aldrich, HPLC grade): phosphate-buffered saline (PBS, pH 7.4) (9:1, 8:2, 7:3, 6:4, 5:5, *v*:*v*). The developing distance was 10 cm. The plates were developed in a closed chromatographic tank, dried at room temperature, and the spots were located under UV light. Every RPTLC analysis was done in triplicate under the same experimental conditions (temperature and humidity). The retention factors (R_f_) were calculated as average values, and the retention constants (R_M_) values were calculated according to R_M_ = log_10_ [1/(R_f_ − 1)]. The calculated R_M_ was plotted versus the volume fraction of MeOH (%MeOH) in the mobile phase, and the R_M_^0^ was the intercept (0% MeOH) of the linear equation R_M_ = R_M_^0^ − b% MeOH.

### 3.3. 4-Chloro-6,7-bis(2-methoxyethoxy)quinazoline logD_7.4_ Determination [36]

A PBS (pH 7.4) solution was saturated with *n*-octanol, and *n*-octanol was saturated with PBS (pH 7.4) by vigorous shaking, followed by equilibration for at least 24 h to ensure complete phase separation. The reference compound 4-chloro-6,7-bis(2-methoxyethoxy)quinazoline and analogs **1**–**7** were dissolved in the octanol phase (previously saturated with PBS), and equal volumes of the aqueous phase (previously saturated with octanol) were added. Final assay solutions were prepared at 10 μM by diluting 2 μL of a 5 mM stock solution into 1 mL of total volume. The biphasic mixtures were shaken for 24 h at room temperature. After equilibration, both the octanolic and aqueous phases were analyzed by UV–visible spectrophotometry using a Shimadzu UV-1603. Compound-specific wavelengths (λ_max_) were employed as follows: **1**, 331 nm; **2**, 332 nm; **3**, 364 nm; **4**, 332 nm; **5**, 313 nm; **6**, 322 nm; **7**, 321 nm; **erlotinib**, 332 nm; and **4-chloro-6,7-bis(2-methoxyethoxy)quinazoline**, 348 nm. LogD_7.4_ values were calculated as log_10_(absorbance_buffer_/absorbance*_n_*_-octanol_). Each determination was performed in triplicate. Regression analyses and curve fittings were performed using GraphPad Prism 8.0 software (GraphPad Software Inc., LaJolla, CA, USA).

### 3.4. Quantitative Structure–Activity Relationship Studies

The dependent variables were the -log_10_ of the corresponding biological IC_50_ values. Experimental physicochemical descriptors, related to lipophilicity (R_M_^0^, see above) and electronics (^1^H NMR chemical shifts), were used for the analyses as independent variables.

The relationships between the selected descriptors and the activities were analyzed by the use of simple and multiple linear regressions. In the equations, *r* is the square root of the correlation coefficient, *r*_adj_ is the square root of the adjusted correlation coefficient, *RSS* is the residual sum of squares, and the *F* value is related to the F-statistical analysis (Fisher test). Regression analyses were performed using GraphPad Prism 8.0 software (GraphPad Software Inc.).

## 4. Conclusions

Our findings demonstrated that lipophilicity plays an important role in modulating the cytotoxicity against glioma cells of the studied series of carborane-containing **Erl** analogs. On the other hand, the boron-cluster electronic effects on these activities should not be dismissed. The role of both lipophilicity and electronic effect on the inhibition of various EGFR isoforms should be explored in greater depth in the future by expanding the population of carborane-containing compounds under study. To further advance this research, future efforts should also include testing a broader range of analogs, assessing in vivo efficacy in glioma models, and extending in silico analyses to rationalize interactions with different EGFR isoforms and mutants.

Overall, these findings highlight the importance of precisely tuning the physicochemical properties of boron-rich molecules to enhance their biological performance and guide the rational design of next-generation anticancer agents.

## Figures and Tables

**Table 1:** Previous biological results [19] of the **Erl** analogs, **1**–**7**, studied herein.

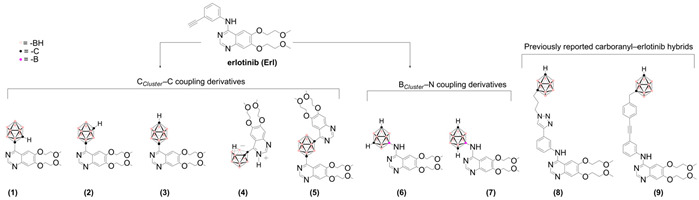						
Compd.	IC_50_ (µM) ^1^					
	U87 MG	F98	C6	Glial Cell ^2^	EGFR_wt_	EGFR_T790M_
**1**	32.5	54.8	11.6	60.4	1.81	NDR ^3^
**2**	36.0	24.6	24.9	51.0	NDR	NDR
**3**	22.0	24.2	24.3	50.0	12.13	12.31
**4**	>100	50.1	>100	>100	NDR	8.73
**5**	>100	24.2	24.8	>100	>5.0	NDR
**6**	47.0	75.5	35.4	71.2	9.19	NDR
**7**	25.4	15.6	8.50	58.5	9.23	7.19
**8**	70	ND	30	>100	0.002	1.13
**9**	ND	ND	>100		517.2	NDR
**Erl**	63.0	>100	>100	>100	0.057	NDR

^1^ The errors were omitted to simplify the visualization. See the original publication for complete information [19]. ^2^ Mix of primary glial cell culture. ^3^ NDR: No dose–response relationship observed within the tested concentration range; ND: not determined. Compounds **8** and **9**, previously reported in reference [13], were included in this study to expand the set of analyzed derivatives in selected experiments.

**Table 2:** Lipophilicities (R_M_^0^ and flog*P*) and independent variable: lipophilic fragmentary values of carboranyl substituents and NMR-chemical shifts of selected protons for the studied compounds.

Compd.	Descriptor				
	R_M_^0^	log_10_R_M_^0^	flog*P* ^1^	*π* _substituent_ ^2^	δ_H_ (ppm) ^3^
**1**	2.46	0.391	4.96	*o*-carboran-1-yl, +4.33	5.71
**2**	3.36	0.526	4.92	*m*-carboran-1-yl, +4.29	3.33
**3**	3.62	0.559	5.10	*p*-carboran-1-yl, +4.47	3.00
**4**	1.46	0.164	-	-	2.41
**5**	3.94	0.600	5.56	*m*-carboran-1-yl, +4.29	-
**6**	2.07	0.316	2.64	*m*-carboran-9-yl, +3.20	2.90
**7**	2.95	0.470	3.48	*p*-carboran-2-yl, +4.04	2.74
**8**	-	-	7.73	-	-
**9**	-	-	9.01	-	-
**Erl**	2.67	0.427	2.07	-	-

^1^ flog*P* was calculated, for example, for **1**, **2**, **3**, **6**, **7** and **Erl**, as log*P*_4-chloro-6,7-bis(2-methoxyethoxy)quinazoline_ − π_Cl_ + (*π*_NH2_)_0 or 1_ + π_substituent_. ^2^ From references [26,27]. ^3^ Chemical shift of the proton bound to the carbon atom located at the vertex of the boron clusters. The fragment lipophilicity of compounds **8** and **9** was determined as follows: Compound **8**: fLog*P* = 2.07 − 0.48 (alkyne) + 0.40 (triazole) + 1.45 (propyl) + 4.29 (carborane) = 7.73. Compound **9**: fLog*P* = 2.07 + 2.15 (phenyl) + 0.50 (methylene) + 4.29 (carborane) = 9.01.

## Data Availability

All data supporting the findings of this study are contained within the article.

## Abbreviations

The following abbreviations are used in this manuscript:

TKR	Tyrosine Kinase Receptor
EGFR	Epidermal Growth Factor Receptor
PDGF	Platelet-Derived Growth Factors
HER2	Human Epidermal Growth Factor 2
Erl	Erlotinib
Sun	Sunitinib
Lap	Lapatinib
BNCT	Boron Neutron Capture Therapy
NDR	No Dose-Response
RPTLC	Reverse Phase Thin-Layer Chromatography
RPHPLC	Reverse Phase High Performance Liquid Chromatography
